# Erector Spinae Plane Block in Abdominal Surgery: A Meta-Analysis

**DOI:** 10.3389/fmed.2022.812531

**Published:** 2022-02-23

**Authors:** Dmitriy Viderman, Mina Aubakirova, Yerkin G. Abdildin

**Affiliations:** ^1^Department of Biomedical Sciences, Nazarbayev University School of Medicine, Nur-Sultan, Kazakhstan; ^2^Department of Mechanical and Aerospace Engineering, School of Engineering and Digital Sciences, Nazarbayev University, Nur-Sultan, Kazakhstan

**Keywords:** regional anesthesia, erector spinae plane block, abdominal surgery, pain management, postoperative analgesia, opioid consumption

## Abstract

**Background:**

Abdominal surgery is one of the most definitive and mainstay treatment options for abdominal pathologies in clinical practice. Acute postoperative pain is a major challenge in the postoperative period. Although opioids are commonly used for analgesia after major abdominal surgeries, they can lead to side effects, such as nausea and vomiting, constipation, pruritus, and life-threatening respiratory depression. Regional anesthetic techniques are commonly used to prevent or minimize these side effects. The objective of this meta-analysis is to assess the effectiveness of erector spinae plane block (ESPB) and standard medical (no block) pain management after major abdominal surgeries.

**Methods:**

We searched for articles reporting the results of randomized controlled trials on ESPB and no block in pain control published before May 2021.

**Results:**

The systematic search initially yielded 56 publications, 49 articles were excluded, and seven randomized clinical trials were included and analyzed. We extracted the data on postoperative opioid consumption, the efficacy of pain relief, time to the first opioid demand, and the rate of postoperative complications in the ESPB group and no block group.

**Conclusions:**

Opioid requirement and time to first analgesic request were significantly reduced in the ultrasound-guided ESPB group, but pain scores, nausea, and vomiting did not differ significantly after pooling the results of the block and no block studies. There were no reports on serious complications related to ESPB.

## Introduction

Abdominal surgery is a common and definitive treatment option for abdominal pathologies in clinical practice. Acute postoperative pain is a major challenge in the postoperative period and is often not optimized. Inadequate perioperative pain management may result in complications such as delayed mobilization, nausea, ileus, prolonged hospital stays, and the development of chronic pain syndromes (1). A U.S. National Institutes of Health report demonstrated that fewer than 50% of patients received adequate pain relief and more than 80% of patients suffer from postoperative pain (2). A series of consecutive reports from the United States (1993, 2003, and 2012) demonstrated that postoperative pain management and the quality of perceived pain have remained mostly unchanged (3). Currently, opioids are one of the most commonly used analgesics for perioperative pain control after major abdominal surgeries (4). Although opioids can provide effective pain relief, they can result in side effects, including nausea and vomiting, constipation, pruritus, and life-threatening respiratory depression (4).

Therefore, to prevent or minimize these side effects, regional anesthetic techniques are commonly added to postoperative pain management (5).

Although epidural anesthesia can provide effective pain control after major abdominal surgeries, postoperative hypotension caused by epidural block, and hypocoagulability are frequent concerns (6).

Regional interfascial plane blocks recently introduced into clinical practice can improve the quality of postoperative pain control. Erector spinae plane block (ESPB) was first performed and described by Forero “as a successful interfascial plane block for thoracic neuropathic pain” (7) and is now one of the most frequently studied types of plane blocks.

ESPB is performed by administering local anesthetic in the erector spinae plane and produces an effective and wide sensory block in the ipsilateral thorax (7).

ESPB has been shown its value and efficacy in acute as well as in chronic pain management (8, 9).

Since the number of ESPB performed for postoperative analgesia in recent years is increasing rapidly, we decided to conduct a systematic review.

The primary objective of this systematic review and meta-analysis is to compare postoperative opioid consumption between ESPB and no block. The secondary objectives include the efficacy of pain relief, time to the first opioid demand, and the rate of postoperative nausea and vomiting.

## Methods

### Protocol

We developed a protocol with the inclusion and exclusion criteria for relevant articles. The method of analysis was established and approved by all authors. This is a meta-analysis investigating ESPB vs. No block in patients who underwent abdominal surgeries. This quantitative systematic review was conducted in accordance with the PRISMA guidelines.

### Search Methods

Using PubMed, Google Scholar, and the Cochrane Library, we conducted a search for relevant articles available in these databases from their inception to May 2021. The search terms included the combination of “erector spinae plane block,” “erector spinae block,” “ESP block,” “ESPB” AND “abdominal surgery,” “abdominal cancer surgery.” We searched the journals and references for all articles relevant to the study. Neither ethical approval nor patient consent was required.

**Inclusion Criteria:**

1) Randomized controlled trials (RCT);
2) Age—18 years old and older;
3) ESPB (bilateral single shot) in acute pain management after abdominal surgery and no block pain management methods assessed using the standard scales, VAS (visual analog pain score) and NRS (numerical pain rating score).
4) Both open and laparoscopic abdominal surgery.

**Exclusion Criteria:**

1) Non-RCTs: case reports or series, editorials
2) Cadaver studies, retrospective studies, technical reports.

**Patient, Intervention, Comparison, and Outcomes (PICO) Criteria:**

We included studies that fit the following criteria:

Population: Adults (>18 years) undergoing abdominal surgeries;

Intervention: bilateral erector spinae plane blocks for abdominal surgeries (ESPB);

Comparator: No block or placebo (sham);

Outcomes:

Primary—to compare ESPB and no block in opioid consumption during the first 24 h following surgery;

Secondary—comparison of pain scores after surgery (VAS, NRS); time to first rescue opioid administration; side effects of opioids (nausea and vomiting); side effects and complications related to ESPB including local anesthetic systemic toxicity (LAST).

**Studies to be included**: randomized controlled clinical trials.

### Types of Interventions

Ultrasound-guided ESPB was performed in the interventional group, whereas the control group received a placebo or “no block”.

### Types of Comparisons

Comparisons between the groups were made. The main outcome of the meta-analysis was to compare opioid consumption during the first 24 h following surgery. The secondary objectives included comparing pain scores after surgery, time to first rescue opioid administration, side effects of opioids (nausea and vomiting), and other complications if any.

Three reviewers independently screened the titles and abstracts to identify articles that meet the review inclusion criteria.

### Data Extraction and Statistical Methods

We extracted and entered data in a systematic review data table. The extracted data included the following rubrics: authors, reference, year of publication, surgery type, sample size, time of the block, and adverse events (as shown in the Table 1).

**Table 1:** Characteristics of the included studies.

**References**	**Country**	**Study design**	**Primary, secondary outcomes**	***N* of patients [total (i./c)]**	**Age (i./c, mean ± SD)**	**Group**	**Surgery**	**ASA**	**T of pain score**	**GA**	**Anesthetic and concentration**	**Postoperative analgesia**	**Conclusions**
Abdelhamid et al. (15)	Egypt	RCT	Prim. – pain scores Sec. – PO opioid consumption	66 (22/22/22)	37.1 ± 10.4 35.9 ± 8.8 35.7 ± 8.6	ESPB: US-guided bilateral ESPB TAP: Bilateral TAP C: Opioid analgesia	Sleeve gastrectomy in obese patients	II/III	0.5, 2, 4, 6, 8, 12, 18, 24 h	Yes	ESPB: 30 ml 0.25% bupivacaine TAP: 30 ml 0.25% bupivacaine	IV paracetamol 1 g, max 4 g in 24 h at VAS ≥ 3 IV pethidine 50 mg at VAS ≥ 5	Lower pain scores in ESPB group than in TAP and Control groups
Abu Elyazed et al. (16)	Egypt	RCT	Prim. – pain scores at 2 h PO Sec. – pain scores at rest up to 24 h, use of intraoperative fentanyl and rescue analgesia in 24 h PO	60 (30/30)	42.7 ± 8 44.3 ± 9.3	ESPB: US-guided bilateral ESPB C: Placebo	Open epigastric hernia repair	I/II	0.5, 1, 2, 4, 6, 8, 12, 18, 24 h	Yes	ESPB: 20 ml bupivacaine 0.25% C: 1 ml of normal saline	IV paracetamol 1 g every 6 h IV pethidine 0.5 mg/kg at VAS ≥ 4	Lower PO pain scores and use of intraoperative fentanyl and PO rescue analgesia in ESPB group
Hamed et al. (17)	Egypt	RCT	Prim. – fentanyl use in 24 h PO Sec. – pain scores, hospital LoS, complication	60 (30/30)	50.00 ± 5.7 50.7 ± 4.72	ESPB: US-guided ESPB C: Placebo	Total abdominal hysterectomy	I / II/ III	0.5, 2, 4, 6, 12, 24 h	Yes	ESPB: 20 ml bupivacaine 0.5% C: 20 ml saline 0.9%	PCA: fentanyl Oral acetaminophen 1 g 4 times daily	Lower fentanyl consumption and pain scores in ESPB group
Kamel et al. (18)	Egypt	RCT	Prim. – pain scores, morphine consumption in 24 h PO, time to rescue analgesic Sec. – patient satisfaction, adverse effects	48 (24/24)	53.7 ± 6.5 56.4 ± 5.9	ESPB: US-guided bilateral ESPB TAP: US-guided bilateral TAP	Open total abdominal hysterectomy	I/II	0.5, 2, 4, 6, 8, 12, 16, 20, 24 h	Yes	ESPB: 20 ml bupivacaine 0.375% + 5 ug/ml adrenaline 1:200,000 on each side TAP: 20 ml bupivacaine 0.375% + 5 ug/ml adrenaline 1:200,000 on each side	Rescue analgesia: IV morphine 3 mg at VAS > 3 Pethidine 1 mg/kg every 4 h, max 300 mg daily	Lower pain scores, longer duration of analgesia, and decreased morphine consumption in ESPB compared to TAP group
Kim et al. (19)	Korea	RCT	Prim. – opioid use in 24 h PO Sec. – consumption of rescue analgesia, pain scores	70 (35/35)	57.8 ± 10.0 56.6 ± 10.2	ESPB: US-guided bilateral ESPB C: No block	Laparoscopic liver resection	I/II	1, 6, 12, 24, 48, 72 h	Yes	ESPB: 40 ml of ropivacaine 0.5%	IV morphine 5 mg IV-PCA: fentanyl 15 μg/ml^−1^ with normal saline, at 1 ml/h^−1^, bolus 1 ml, 15 min lockout at NRS > 3	No significant difference in pain scores between ESPB and control group
												Fentanyl 0.5 μg/kg^−1^ bolus at NRS > 4 Ward: IV ibuprofen 400 mg every 6 h on PO day 1 and 2, max 6 doses + IV hydromorphone 1 mg at NRS > 4 PO day 2: Oral codeine phosphate 10 mg/ibuprofen 200 mg/paracetamol 250 mg) every 8 h	
Prasad et al. (20)	India	RCT	Prim. – pain scores Sec. – hemodynamic outcomes	61 (31/30)	41.03 ± 12.58 37.37 ± 16.81	ESPB: ESPB under fluoroscopy guidance C: No block	Percutaneous nephrolithotomy	I/II	1, 2, 3, 4, 6, 12, 18, 24 h	Yes	20 ml 0.375% ropivacaine	IV paracetamol 1 g every 8 h Rescue analgesia: tramadol 2 mg/kg at VAS > 4, max 4 doses in 24 h	More effective postoperative pain relief in ESPB group
Tulgar et al. (21)	Turkey	RCT	Prim. – pain scores at rest and coughing for 24 h PO Sec. – analgesia consumption in 24 h	30 (15/15)	53.6 ± 12.5 50.4 ± 11.2	ESPB: US-guided bilateral ESPB C: No block	Laparoscopic cholecystectomy	I/II	20, 40 min, 1, 3, 6, 12, 18, 24 h	Yes	ESPB: 20 ml of 0.375% bupivacaine bilaterally	PCA: tramadol 3 mg/kg (total volumen 100 ml), no basal infusión, 10 mg bolus, 20 min lockout Rescue analgesia: fentanyl 25 μg at NRS ≥ 4 every 20 min Ward: paracetamol 1 g every 8 h + diclofenac Na 75 mg IM at NRS ≥ 4 + meperidine 50 mg IV if pain remained after 1 h	Decreased immediate PO pain and lower rescue analgesia requirement in 12 h in ESPB group

*ASA status, American Society of Anesthesiologists; C, control; ESPB, Erector Spinae Plane Block; GA, general anesthesia; i., intervention; IM, intramuscular(ly); IV, intravenous(ly); LoS, length of stay; max, maximum; N, number, NRS, Numeric Rating Scale; PCA, patient-controlled analgesia; PO, postoperative(ly); prim., primary; RCT, randomized controlled trial; SD, standard deviation; sec., secondary; T, time; TAP, transversus abdominis plane block; US, ultrasound; VAS, Visual Analogue Scale*.

If data were given in terms of median and interquartile range, the mean and SD were recalculated using the following approach described by Luo et al. (10) (sample mean) and Wan et al. (11) (sample SD).

Postoperative opioid doses were converted to intravenous morphine equivalents (mg) (12–14). We conducted the data analysis using the Review Manager (RevMan) [Computer program]. Version 5.4. The Cochrane Collaboration, 2020. Statistical heterogeneity was calculated using the I^2^ statistic.

## Results

### Description of Included Studies

**Figure 1:**
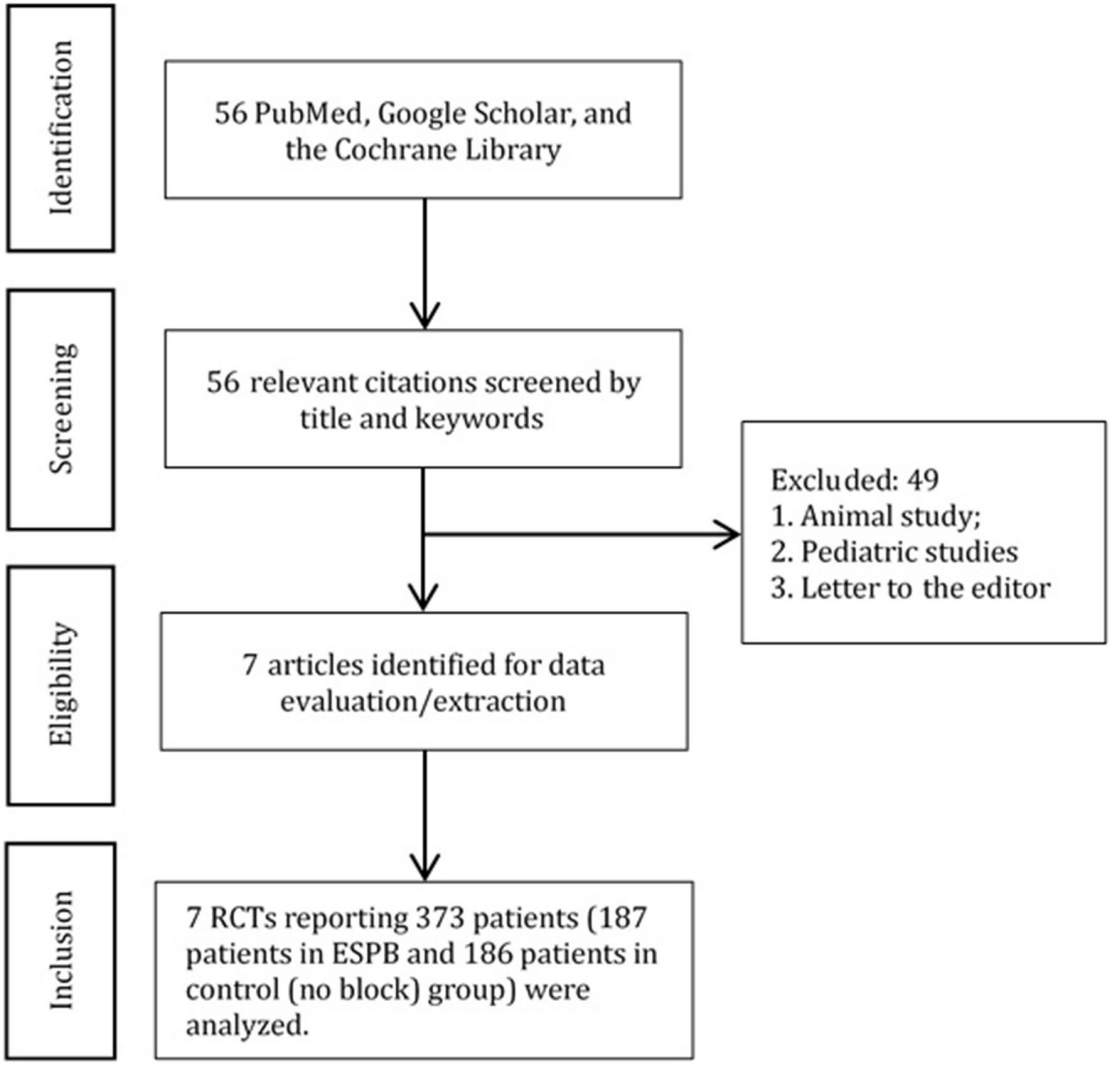
PRISMA diagram.

The systematic search initially yielded 56 publications, of which 49 articles were excluded. A total of 373 patients [187 patients in ESPB and 186 patients in the control (no block) group] undergoing abdominal surgeries from 7 randomized clinical trials were analyzed (Figure 1). The data and characteristics of the included studies are presented in Table 1.

#### Total Opioid Consumption Within 24 h After Surgery (mg)

**Figure 2:**
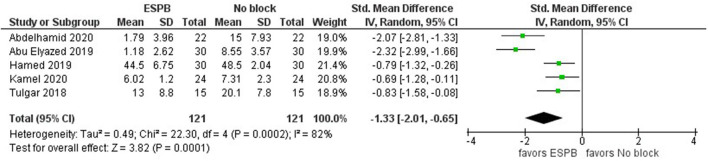
Forest plot of total opioid consumption for the ESPB vs. non-block care studies in the first 24 h after surgery (in mg of morphine).

The results showed that five studies analyzed the total opioid consumption within 24 h after surgery in the ESPB and control (no block) groups. In particular, the studies reported the total pethidine consumption (15, 16), the total fentanyl consumption (17), the total morphine consumption (18), and the total tramadol consumption (19). The former two did not report the sample means and sample standard deviations; therefore, we estimated them as discussed in the pertinent literature (10, 11). As shown in Figure 2, the meta-analysis favors ESPB over control (no block) because all studies report lower opioid consumption in the ESPB groups than in the control groups; the standardized mean difference with 95 CI is −1.33 [−2.01,−0.65]. We calculated opioid consumption in terms of morphine (mg) consumption as follows: pethidine (mg) and tramadol (mg) values were multiplied by 0.1, while fentanyl (mg) values by 0.01. In other words, fentanyl (mcg) values were converted to morphine (mg) values by multiplying them by 0.1 since 1 mg of morphine is considered equivalent to 10 mcg of fentanyl (https://www.mdanderson.org/) (Figure 2).

For the total opioid consumption meta-analysis, we used the random-effects model with a standardized mean difference since the studies reported the use of multiple different opioids. The study heterogeneity is high (I^2^ = 82%) and that is significant (*P*-value = 0.0002). The total number of individuals in each group is 121. In addition to the above five studies, Prasad et al. (20) found that the tramadol consumption in the ESPB group (*n* = 31, mean = 100 mg) was lower than in the control group (*n* = 30, mean = 350 mg); however, this study was not included into our meta-analysis because it did not report the sample standard deviation for the ESPB group.

#### Pain Severity at 24 h After Surgery

**Figure 3:**
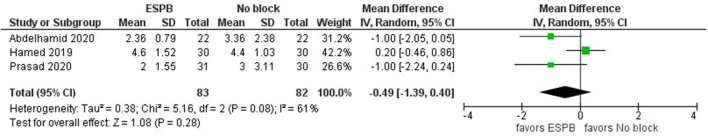
Forest plot of pain intensity in the first 24 h after surgery (in VAS scores).

Based on the results of three studies (15, 17, 20), we build a forest plot on pain severity for ESPB vs. no block (Figure 3). For two of these studies (conducted in 2020), we had to estimate the sample means and sample standard deviations as discussed previously. As shown in Figure 3, the total number of patients in these three studies is 165 (with 83 being in the ESPB groups and the other 82 individuals being in the control groups). The first and third studies tend to favor ESPB over control (because the mean value of pain severity in these studies shows up smaller in ESPB groups). Overall, the forest plot does not favor ESPB over no block, but more studies are required to obtain a clearer result.

#### The First Request for Rescue Analgesia (in Hours)

**Figure 4:**
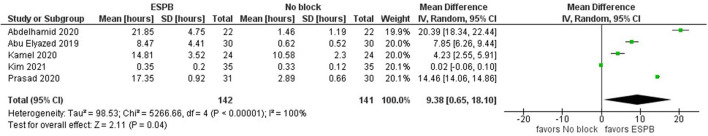
Forest plot of the first request for rescue analgesia (in hours).

The results further showed that five studies compared the time to first request for rescue analgesia between ESPB and control groups (Figure 4) (15, 16, 18–20). Here, we prefer the mean difference since all studies use the same unit (hours) and outcome (continuous). Since the mean differences between ESPB and control groups are positive, the meta-analysis favors ESPB over no block interventions. For two studies (15, 16), the sample means and sample standard deviations were estimated from their medians, first and third quartiles, and sample sizes.

#### Postoperative Complications: Nausea and Vomiting

**Figure 5:**
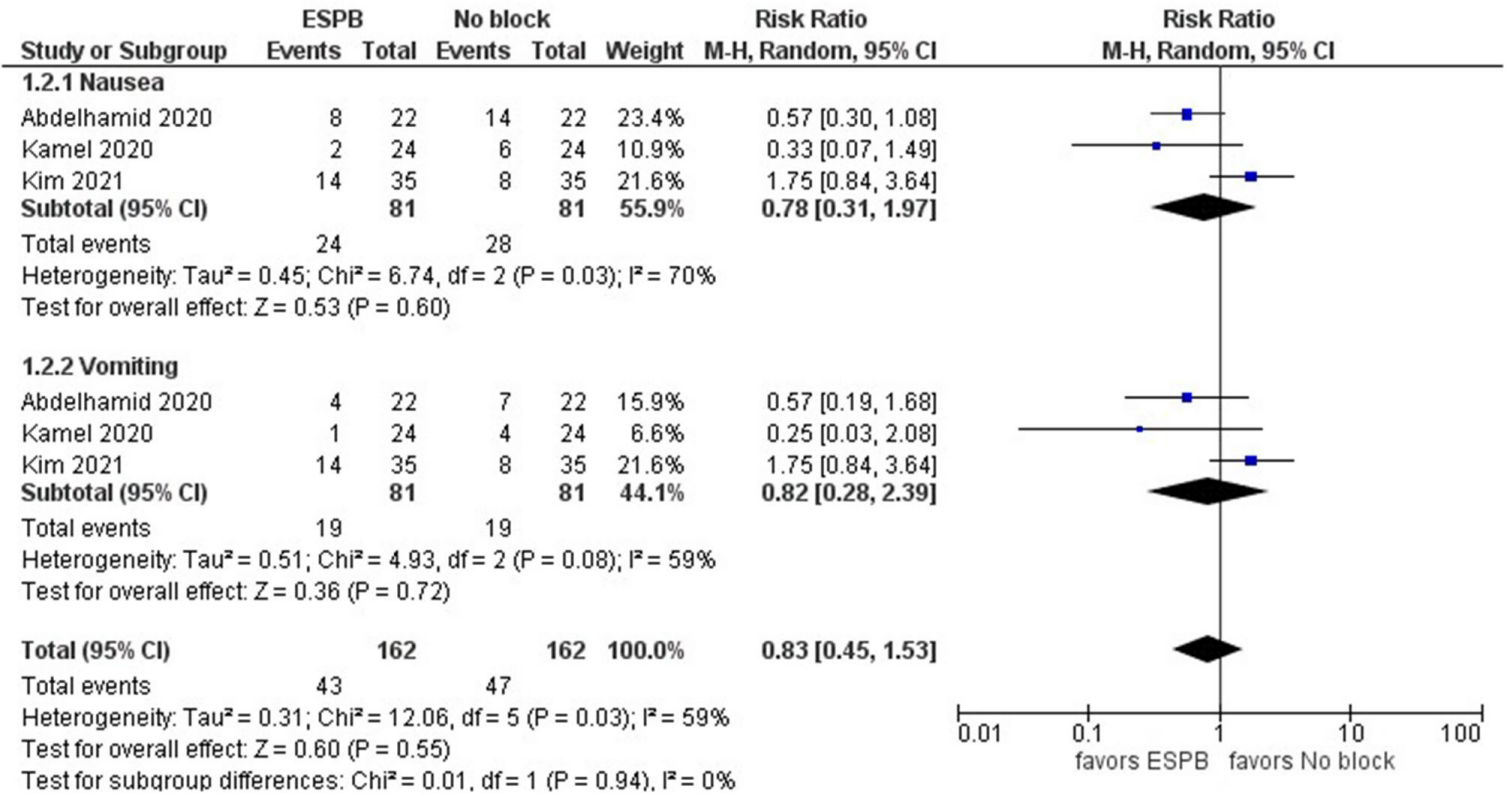
Forest plot of postoperative nausea and vomiting.

Three studies (15, 18, 19) assessed the rates of postoperative nausea and vomiting (PONV). The model shows no difference between ESPB and no block in PONV (Figure 5). The result is insensitive to the exclusion of any study. The risk ratio with 95% CI is 0.83 [0.45, 1.53]. In addition, the first two studies presented the data in shares of people with nausea and vomiting in the samples. In contrast, the latter presented data on the number of patients having postoperative complications and did not separate them into the exact number of individuals having nausea and vomiting. Since they reported the number of patients that developed “postoperative nausea and vomiting 24 h after surgery” as 14 and 8 in ESPB and control groups, respectively, we assume these patients have nausea and vomiting. Note that even if we replace AND with OR, it is unlikely that the conclusion will change. However, it is clear that more studies are required to draw a conclusion of the advantages of ESPB over no block option concerning postoperative complications.

## Discussion

Our meta-analysis analyzed the pain control efficacy of erector spinae plane block after abdominal surgery. Opioid consumption during the first 24 h postoperation was lower in ESPB compared to no block.

Erector spinae plane block (ESPB) did not show a significant pain score reduction (VAS/NRS) at the first 24 h following the surgical procedure. Another finding is that ESPB was better than no block in assessing the time to first demand rescue analgesia. Finally, ESPB was not better than no block in reducing the rates of postoperative nausea and vomiting.

Several recent clinical trials and systematic reviews demonstrated the positive effect of ESPB in non-abdominal surgeries in reducing early postoperative opioid requirement and time to first opioid requirement (22). ESPB can reduce opioid requirement but does not appear to influence opioid-related side effects nor does it improve the quality of pain. One interpretation might be because the ESPB groups also have access to opioids to improve pain intensity further, yet may expose participants to opioid-related side effects such as nausea and vomiting which do not appear to be dose-related, i.e., that the smaller amounts of opioids in the ESPB group are still causing similar amounts of nausea and vomiting.

The following drugs and methods are usually used for postoperative pain control: non-steroidal anti-inflammatory drugs with opioids, and regional techniques, such as ESPB. Combining these drugs can lead to an additive effect increasing the effect of individual analgesic agents and reducing their side effects. Unfortunately, in many cases, drug therapy is insufficient to maintain appropriate postoperative analgesia; therefore, regional techniques, such as ESPB, are commonly used.

Previous trials and our meta-analysis have demonstrated that ESPB reduced postoperative opioid requirements (22). Although opioids possess strong analgesic properties, they delay early mobilization as well as discharge from the hospital, which is a significant drawback in hospitals with high patient volume (23). The rationale of using opioid-sparing pain management techniques, especially in cancer surgery, is based on several considerations.

The current opioid crisis requires new approaches allowing the reduction of opioid use. Moreover, despite the fact that opioids have been shown to be one of the most effective approaches for pain management, the majority of patients report incomplete pain relief and side effects after surgery. Laparotomy is known to induce intense nociceptive stimulation by surgical injury to the skin, parietal and visceral peritoneum, and visceral structures (7).

Erector spinae plane block (ESPB) is a type of facial plane block in which local anesthetic is administered in the plane located between the erector spinae muscle and thoracic transverse process (7). ESPB blocks the transmission of nociceptive stimuli through the dorsal/ventral rami of the spinal nerve roots, prevents afferent stimuli transmission, and inhibits efferent activation of the sympathetic nervous system (7). The effect of ESPB is also achieved through the block of the lateral, posterior, and anterior thoracic wall resulting in multiple level sensory blocks (24). Additional proposed mechanism of action could be explained by the epidural spread of the anesthetics (25).

No major complications related to ESPB were reported. More trials and data on safety and complications related to ESPB are needed (26). Although ESPB might be associated with lower risks of mechanical complications, for example, compared with epidural anesthesia or paravertebral analgesia, because the efficacy of ESBP is volume depended, the risk of intravascular injection and local anesthetic toxicity (LAST) must be considered (27).

ESPB has low risks of complications associated with hypocoagulable states, which might be an issue for epidural anesthesia. Because there are no major vessels in close proximity to the erector spinae plane, the risks of intravascular injections of local anesthetics or hematoma are lower than in other regional anesthetic blocks (27).

Nevertheless, like other types of interfascial plane blocks, especially if high volumes of local anesthetics are used, LAST recognition and management algorithms should be exercised (28). For example, both surgeons and anesthesiologists must be aware of LAST, whenever ESPB is used; all members of the anesthesia and surgical team should maintain good communication (e.g., the surgical team should be informed that high volumes of local anesthetics were used, and the surgical team should be careful in using additional volumes for local infiltration) (28). Since different concentrations of local anesthetics are currently used, it is important to double-check it before injecting (28). If the continuous ESPB is used, telemetry monitoring could be recommended to improve patient safety (28).

The use of lower concentration and high volume solutions was proposed to minimize the risk of LAST; a higher volume of local anesthetics improves the efficacy of the block by covering more segments, whereas low volume theoretically reduces the risks of LAST (29). The ideal concentration and volume of local anesthetics are yet to be investigated.

Although the use of low local anesthetic concentration can reduce the risks of LAST, more opioid anesthetic might be required to provide sufficient pain control. It can, in turn, increase the risks of opioid-related side effects (nausea, vomiting, intestinal hypomotility, and respiratory depression). The incidence of nausea and vomiting after surgery was reported to be significantly lower in the group with high bupivacaine concentration, possibly due to reduced tramadol administration (29).

## Limitations and Future Research

A major limitation of this meta-analysis is that there was a limited number of matched clinical trials focused on ESPB in pain control after abdominal surgeries. The lack of significance is then attributed to inadequate amounts of studies included in the analysis. The second limitation is due to the heterogeneity of the structure, style, and rubrics of the studied and published results. The next major limitation is high heterogeneity in types and extends of surgeries (e.g., gastrectomy, liver resection, hysterectomy, epigastric hernia repair, and cholecystectomy) and approaches (open, laparoscopic). Another limitation is that our literature search might have failed to find all publications related to the review objectives.

## Conclusions

This meta-analysis demonstrated that opioid requirement and time to first analgesic request was significantly reduced in the ultrasound-guided ESPB group, but pain scores, nausea, and vomiting did not differ significantly after pooling the results of the block and no block studies. No serious complications related to ESPB were reported. Future RCTs with more standardized reporting rubrics are warranted to verify and consolidate the results of previous RCTs and this meta-analysis.

## Data Availability Statement

The original contributions presented in the study are included in the article/supplementary material, further inquiries can be directed to the corresponding author/s.

## Author Contributions

DV: protocol, screening, writing, revision, and final approval. YA: statistical analysis, writing, and final approval. MA: data extraction, revision, and final approval. All authors contributed to the article and approved the submitted version.

## Funding

This work was supported by the Nazarbayev University Faculty Development Competitive Research Grant 2021-2023. Funder project reference: 021220FD2851.

## Conflict of Interest

The authors declare that the research was conducted in the absence of any commercial or financial relationships that could be construed as a potential conflict of interest.

## Publisher's Note

All claims expressed in this article are solely those of the authors and do not necessarily represent those of their affiliated organizations, or those of the publisher, the editors and the reviewers. Any product that may be evaluated in this article, or claim that may be made by its manufacturer, is not guaranteed or endorsed by the publisher.

## References

[1] Gottschalk A Durieux ME Nemergut EC. Intraoperative methadone improves postoperative pain control in patients undergoing complex spine surgery. Anesth Analg. (2011) 112:218-23. 10.1213/ANE.0b013e3181d8a095 20418538

[2] Rawal N. Current issues in postoperative pain management. Eur J Anaesthesiol. (2016) 33:160-71. 10.1097/EJA.0000000000000366 26509324

[3] Front Matter. In: *Principles and Practice of Pharmacology for Anaesthetists*. John Wiley and Sons, Ltd (2009). p. i-vii.31721144

[4] Ohkura Y Shindoh J Ueno M Iizuka T Haruta S Udagawa H. A new postoperative pain management (intravenous acetaminophen: Acelio®) leads to enhanced recovery after esophagectomy: a propensity score-matched analysis. Surg Today. (2018) 48:502-9. 10.1007/s00595-017-1616-5 29234960

[5] Crumley S Schraag S. The role of local anaesthetic techniques in ERAS protocols for thoracic surgery. J Thorac Dis. (2018) 10:1998-2004. 10.21037/jtd.2018.02.48 29707356 PMC5906279

[6] Boisen ML Sardesai MP Kolarczyk L Rao VK Owsiak CP Gelzinis TA. The year in thoracic anesthesia: selected highlights from 2017. J Cardiothorac Vasc Anesth. (2018) 32:1556-69. 10.1053/j.jvca.2018.03.001 29655515

[7] Forero M Adhikary SD Lopez H Tsui C Chin KJ. The erector spinae plane block: a novel analgesic technique in thoracic neuropathic pain. Reg Anesth Pain Med. (2016) 41:621-7. 10.1097/AAP.0000000000000451 27501016

[8] Viderman D Dautova A Sarria-Santamera A. Erector spinae plane block in acute interventional pain management: a systematic review. Scand J Pain. (2021) 21:671-9. 10.1515/sjpain-2020-0171 33984888

[9] Viderman D Sarria-Santamera A. Erector spinae plane block in chronic pain management: a scoping review. Tumori. (2021) 107:458-67. 10.1177/0300891620985935 33430714

[10] Luo D Wan X Liu J Tong T. Optimally estimating the sample mean from the sample size, median, mid-range, and/or mid-quartile range. Stat Methods Med Res. (2018) 27:1785-805. 10.1177/0962280216669183 27683581

[11] Wan X Wang W Liu J Tong T. Estimating the sample mean and standard deviation from the sample size, median, range and/or interquartile range. BMC Med Res Methodol. (2014) 14:135. 10.1186/1471-2288-14-135 25524443 PMC4383202

[12] Knotkova H Fine PG Portenoy RK. Opioid rotation: the science and the limitations of the equianalgesic dose table. J Pain Symptom Manage. (2009) 38:426-39. 10.1016/j.jpainsymman.2009.06.001 19735903

[13] Mercadante S Caraceni A. Conversion ratios for opioid switching in the treatment of cancer pain: a systematic review. Palliat Med. (2011) 25:504-15. 10.1177/0269216311406577 21708857

[14] Santonocito C Noto A Crimi C Sanfilippo F. Remifentanil-induced postoperative hyperalgesia: current perspectives on mechanisms and therapeutic strategies. Local Reg Anesth. (2018) 11:15-23. 10.2147/LRA.S143618 29670398 PMC5898588

[15] Abdelhamid BM Khaled D Mansour MA Hassan MM. Comparison between the ultrasound-guided erector spinae block and the subcostal approach to the transversus abdominis plane block in obese patients undergoing sleeve gastrectomy: a randomized controlled trial. Minerva Anestesiol. (2020) 86:816-26. 10.23736/S0375-9393.20.14064-1 32449336

[16] Abu Elyazed MM Mostafa SF Abdelghany MS Eid GM. Ultrasound-guided erector spinae plane block in patients undergoing open epigastric hernia repair: a prospective randomized controlled study. Anesth Analg. (2019) 129:235-40. 10.1213/ANE.0000000000004071 30801359

[17] Hamed MA Goda AS Basiony MM Fargaly OS Abdelhady MA. Erector spinae plane block for postoperative analgesia in patients undergoing total abdominal hysterectomy: a randomized controlled study original study. J Pain Res. (2019) 12:1393-8. 10.2147/JPR.S196501 31118757 PMC6503185

[18] Kamel AAF Amin OAI Ibrahem MAM. Bilateral ultrasound-guided erector spinae plane block versus transversus abdominis plane block on postoperative analgesia after total abdominal hysterectomy. Pain Physician. (2020) 23:375-82. 10.36076/ppj.2020/23/375 32709172

[19] Kim D Kim JM Choi GS Heo G Kim GS Jeong JS. Ultrasound-guided erector spinae plane block for postoperative analgesia in laparoscopic liver resection: a prospective, randomised controlled, patient and observer-blinded study. Eur J Anaesthesiol. (2021) 38(Suppl 2):S106-12. 10.1097/EJA.0000000000001475 33653982

[20] Prasad MK Varshney RK Jain P Choudhary AK Khare A Jheetay GS. Postoperative analgesic efficacy of fluoroscopy-guided erector spinae plane block after percutaneous nephrolithotomy (PCNL): a randomized controlled study. Saudi J Anaesth. (2020) 14:480-6. 10.4103/sja.SJA_26_20 33447190 PMC7796763

[21] Tulgar S Kapakli MS Senturk O Selvi O Serifsoy TE Ozer Z. Evaluation of ultrasound-guided erector spinae plane block for postoperative analgesia in laparoscopic cholecystectomy: a prospective, randomized, controlled clinical trial. J Clin Anesth. (2018) 49:101-6. 10.1016/j.jclinane.2018.06.019 29913392

[22] Zhang Y Liu T Zhou Y Yu Y Chen G. Analgesic efficacy and safety of erector spinae plane block in breast cancer surgery: a systematic review and meta-analysis. BMC Anesthesiol. (2021) 21:59. 10.1186/s12871-021-01277-x 33610172 PMC7896394

[23] Wigmore T Farquhar-Smith P. Opioids and cancer: friend or foe? Curr Opin Support Palliat Care. (2016) 10:109-18. 10.1097/SPC.0000000000000208 26990052

[24] Hickey OT Burke SM Hafeez P Mudrakouski AL Hayes ID Shorten GD. Severity of acute pain after breast surgery is associated with the likelihood of subsequently developing persistent pain. Clin J Pain. (2010) 26:556-60. 10.1097/AJP.0b013e3181dee988 20639740

[25] Altiparmak B Korkmaz Toker M Uysal AI Kuşçu Y Gümüş Demirbilek S. Ultrasound-guided erector spinae plane block versus oblique subcostal transversus abdominis plane block for postoperative analgesia of adult patients undergoing laparoscopic cholecystectomy: randomized, controlled trial. J Clin Anesth. (2019) 57:31-6. 10.1016/j.jclinane.2019.03.012 30851501

[26] Temirov T Ben-David B Mustafin A Viderman D. Erector spinae plane block in management of pain after kidney transplantation. Pain Med. (2019) 20:1053-4. 10.1093/pm/pny221 30412258

[27] Chin KJ Chan V. Ultrasound-guided peripheral nerve blockade. Curr Opin Anaesthesiol. (2008) 21:624-31. 10.1097/ACO.0b013e32830815d1 18784490

[28] Viderman D Ben-David B Sarria-Santamera A. Analysis of bupivacaine and ropivacaine-related cardiac arrests in regional anesthesia: a systematic review of case reports. Rev Esp Anestesiol Reanim. (2021) 68:472-83. 10.1016/j.redare.2020.10.005 34538765

[29] Altiparmak B Korkmaz Toker M Uysal AI Gümüş Demirbilek S. Comparison of the efficacy of erector spinae plane block performed with different concentrations of bupivacaine on postoperative analgesia after mastectomy surgery: ramdomized, prospective, double blinded trial. BMC Anesthesiol. (2019) 19:31. 10.1186/s12871-019-0700-3 30832580 PMC6399855

